# Differences in the Behavioral Parameters of Young Zebu and Composite Bulls Kept on Non-Forested or in Integrated Crop–Livestock–Forestry Systems

**DOI:** 10.3390/ani14060944

**Published:** 2024-03-19

**Authors:** Mariana Jucá Moraes, Erick Fonseca de Castilho, Júlio Cesar de Carvalho Balieiro, Alberto Carlos de Campos Bernardi, Andréa do Nascimento Barreto, Lívia Ferreira Pinho, Giovanna Galhardo Ramos, Gabriela Novais Azevedo, Letícia Krügner Zanetti, Alexandre Rossetto Garcia

**Affiliations:** 1 Institute of Veterinary Medicine, Federal University of Pará, Av. dos Universitários, s/n, Castanhal 68746-360, PA, Brazil; marianajmoraes15@gmail.com (M.J.M.); andreadnb91@gmail.com (A.d.N.B.); liviapinho30@gmail.com (L.F.P.); 2 Institute of Animal Health and Production, Federal Rural University of the Amazon, Av. Perimetral, Belém 66077-830, PA, Brazil; erick.castilho@ufra.edu.br; 3 School of Veterinary Medicine and Animal Science, University of São Paulo, Av. Duque de Caxias Norte, Pirassununga 13630-520, SP, Brazil; balieiro@usp.br (J.C.d.C.B.); giiovannagalhardo@gmail.com (G.G.R.); 4 Brazilian Agricultural Research Corporation, Embrapa Southeast Livestock, Rod. Washington Luiz, São Carlos 13560-970, SP, Brazil; alberto.bernardi@embrapa.br; 5 School of Veterinary Medicine, Central University Centre of São Paulo, R. Miguel Petroni, São Carlos 13563-470, SP, Brazil; gabi.nazevedo@hotmail.com (G.N.A.); letyzanettii@gmail.com (L.K.Z.)

**Keywords:** adaptive capacity, animal behavior, cattle management, beef cattle, sustainability, precision livestock farming

## Abstract

**Simple Summary:**

Understanding the principles of beef cattle behavior and their attitudes, whether as a result of the learning process or as a response to environmental stimuli, is important for providing greater sensitivity and efficiency to modern production systems. Our study evaluated the behavior of young zebu (Nelore) and composite (Canchim) bulls kept in pasture production systems, either in a crop–livestock–forest integration (ICLF) or without afforestation. The behavior of animals was assessed electronically and by direct observation. The results revealed that the breed and production system influenced the expression of the animals’ daily activities. The ICLF system had a milder microclimate and favored thermal comfort. Natural shading influenced grazing, resting, and rumination time, but did not interfere with cortisol concentration. Grazing and rumination times were longer for Canchim than Nelore bulls, while Nelore bulls spent more time resting, either standing or lying. The breed had no impact on the frequency of water and mineral mixture intake. This investigation sheds light on the differences in animal behavior depending on their breed and the configuration of production systems, which vary with the availability of natural shade. This knowledge can help producers make decisions, ensuring greater animal welfare, better working conditions, and greater management efficiency.

**Abstract:**

The behavior of ruminants can influence their productive efficiency. The aim of this study was to evaluate the behavior of young zebu and composite bulls kept in pasture production systems, either in a crop-livestock-forest integration or without afforestation. The work was carried out in São Carlos, Brazil (21°57′42″ S, 47°50′28″ W), in a high-altitude tropical climate, from March to July, 2022. Forty young bulls were evaluated, being 20 Nelore (*Bos indicus*) (342.5 ± 36.6 kg BW; 16.9 ± 1.8 months) and 20 Canchim (5/8 *Bos taurus* × 3/8 *Bos indicus*) (338.4 ± 39.8 kg BW; 19.1 ± 1.9 months), equally distributed in full-sun (FS) and integrated crop–livestock–forestry (ICLF) production systems. Behavior was monitored uninterruptedly by an acoustic sensor and accelerometer attached to a collar, and complemented by direct visual assessment, in two one-day campaigns per month. Serum cortisol concentration was assessed monthly. Statistical analyses were conducted using a general linear model at a 5% significance level (SAS, version 9.4). The ICLF system had a milder microclimate and favored thermal comfort. Natural shading influenced grazing, resting, and rumination time. The Canchim bulls were more active when moving and grazing (*p* < 0.05), even at the hottest times of the day. In turn, the Nelore bulls spent more time resting at all times (*p* < 0.001), which was shown to be an adaptive strategy in response to environmental stimuli. The Canchim bulls had a longer rumination time than the Nelore bulls (*p* < 0.001), due to their longer grazing time. The frequency of water and mineral mixture intake did not differ between genotypes, regardless of the production system (*p >* 0.05). There was no difference in the serum cortisol concentrations of the Nelore and Canchim bulls kept in FS or ICLF (*p* = 0.082). Thus, young bulls of the different genotypes showed different behaviors, regardless of whether they were kept on pasture without afforestation or in an integrated crop–livestock–forestry system.

## 1. Introduction

Over the years, several countries have been facing the effects of climate change, which has caused direct damage to the agricultural sector [1]. In livestock farming, beef cattle are heavily impacted by extreme or sudden environmental variations. When raised on pasture, the herds are consistently more exposed to abiotic factors, including the effects of high temperatures and solar radiation. As a result of climate and environmental changes, bovines have been highly susceptible to heat stress and water and feed restrictions [2,3]. Climate change has also directly influenced food security by affecting growth, reproduction, and the resilience of animals to diseases [4,5]. Specifically, heat stress alters physiological responses and normal metabolic state and modifies the animal behavior; that is, the greater the discomfort to which the animal is exposed, the more pronounced are the changes [6,7].

Currently, studies are being carried out to mitigate the impacts of the increased frequency and intensity of heat waves on pasture-based production systems, both in tropical and temperate climate regions [8]. Meanwhile, consumers are becoming increasingly aware of the welfare of farm animals, and this issue has been discussed as a relevant dimension of the sustainability of production systems [9]. In this context, the integrated crop–livestock–forestry system (ICLF) is a highly effective technology for recovering degraded soils and pastures [10], capable of reducing the vulnerability of production systems and increasing animal welfare [11]. Shaded pastures reduce the direct solar radiation load on animals [12] and minimize losses due to heat stress, such as reduced dry matter intake, slower growth, and low weight gain [13,14,15].

Zebu cattle (*Bos taurus indicus*) are an important genetic base for beef production in many tropical countries. Originating from India and widely exploited in several tropical countries due to its high fertility at pasture and adaptability, the Nelore breed stands out among the zebu genotypes with the greatest population and economic expressiveness, [16]. Nelore cattle are particularly adapted to hot climates due to their morphophysiological characteristics, such as pigmented and thin skin, high hair density, white or light gray coat, and short, light, and laid hair [17]. In turn, the Canchim breed is a composite genotype, derived from crossing Zebu and Charolais. The main purpose of its composition was to combine the rusticity and adaptability of zebu with the greater growth speed and carcass quality of the taurine. Based on the best conformation and performance results, the Canchim breed was fixed at a ratio of 5/8 Charolais and 3/8 Zebu [18]. The Canchim breed is characterized by short, laid hair, low hair density, and a bay or bright yellow color [19]. This breed stands out for its high fertility and maternal ability and for its ability to gain weight, both in confinement and on pasture [20].

Ruminants behave in defined patterns, such as grazing, ruminating, and resting, which can influence their productive and reproductive responses [21]. Visual observation of animal behavior is a technique adopted in several scientific studies, with consistent results. However, its use in the assessment of cattle at pasture has some limitations, such as the restriction of application to daytime periods only, and the need for the constant presence of the observer at the production system [22]. For this reason, the use of precision livestock instruments has been gaining ground in animal monitoring studies and the decoding of behavioral patterns [2,23]. Electronic sensors associated with digital control systems allow automatic, continuous, and real-time monitoring of animal activity, production, reproduction, welfare, and health attributes, either when isolated or in a group [24]. These electronic devices have been used successfully on confined cattle [25] and animals kept on pasture [26,27].

Nelore and Canchim animals have different phenotypic characteristics, as they have different genetic compositions. By assumption, the phenotypic differences can imprint specific physiological and metabolic responses on the animals and alter their behavior, especially in the face of challenging environmental conditions [28]. For example, cattle breeds native to tropical areas are highly adapted to direct heat stress and spend more time grazing than resting in the shade. Thus, Zebu cattle have been observed to adapt their grazing behavior in response to limited grazing time [1]. In turn, *Bos taurus* animals stand for longer periods and lie for shorter periods in high ambient temperatures [29]. Therefore, assessing the behavior of animals of different genotypes and in different production systems is of utmost importance for a better understanding of the relationships between individuals and production environments, which may differ in spatial configurations and microclimates. Therefore, this study was based on the hypothesis that young Zebu and composite bulls show similar ethological responses when raised on pasture, with or without natural shading.

In order to expand knowledge about the different behaviors of animals of different breeds raised on pasture under the same microclimatic (sun or shade) and management conditions, the aim of this study was to evaluate the behavioral parameters (movement, rumination, resting, posture, and attitudes) of young bulls of the Nelore and Canchim breeds kept on pastures without or with an integrated crop–livestock–forestry system, as well as to analyze the possible differences in responses between the genotypes, due to the microclimatic conditions provided by the different production systems.

## 2. Material and Methods

### 2.1. Place, Period, and Climate Characterization

The experiment was carried out over 5 months (March to July 2022) at Embrapa, the Brazilian Agricultural Research Corporation in São Carlos, SP, Brazil (21°57′42″ S, 47°50′28″ W, altitude 860 m). The local climate type is Cwa, altitude tropical. Throughout the year, the maximum air temperature ranges from 29.2 to 38.0 °C. The average relative humidity ranges from 55.3 to 90.5%. Average annual rainfall is 1361 mm. Average summer solar radiation is 20.85 MJ/m^2^/day [30].

### 2.2. Bioethics

The experiment was conducted and reported in accordance with ethical principles in animal research. The protocols were previously evaluated by the Ethics Committee for the Use of Experimental Animals at Embrapa Pecuária Sudeste (CEUA PRT 02/2020).

### 2.3. Meteorological Variables and Thermal Comfort Indices

The microclimate was continuously characterized using two automatic weather stations installed within the production systems, one in the full-sun area (FS) and the other in the forested area of the integrated crop–livestock–forestry (ICLF) system. The variables recorded were: air temperature (AT, °C), relative humidity (RH, %), black globe temperature (BGT, °C), and wind speed (WS, m/s). The weather stations were programmed to record every minute and present data outputs every 15 min, throughout the 24 h of the day.

The black globe temperature and humidity index (BGHI) was calculated as proposed by [31], and used as an indicator of animal thermal comfort:

BGHI = BGT + 0.36 (DPT) + 41.5

where BGT = black globe temperature (°C) and DPT = dew point temperature (°C).

The radiant heat load (RHL, W/m^2^) indicates the total amount of radiation received by the animals and was calculated using the model proposed by [32]:

RHL = σ (MRT)^4^

where σ = the Stefan–Boltzman constant (5.67 × 10^−8^ K^−4^ W/m^2^) and MRT = mean radiant temperature (K) for each production system.

$$\mathrm{MRT}=10\sqrt[{4}]{2.51}\times \sqrt{\mathrm{WS}\;}\times \left(\mathrm{BGT}-\mathrm{DBT}\right)+{\left(\frac{\mathrm{BGT}}{100}\right)}^{4}$$

where:

WS = wind speed (m/s), DBT = dry bulb temperature (°C)

### 2.4. Characterization of Production Systems

**Figure 1:**
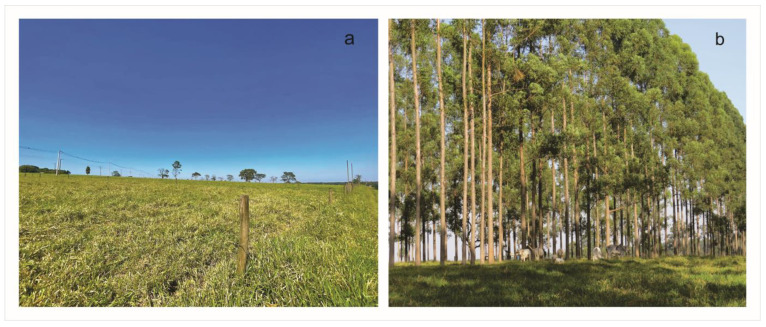
Photographs of the production systems in the experimental area, with *Urochloa brizantha* pastures (**a**) in full-sun (FS) and (**b**) in integrated crop–livestock–forestry (ICLF) systems for beef cattle.

Two production systems (Figure 1) were used in the experiment: (a) a full-sun system (FS)—a pasture system with an area of 12 ha, with 4 subsystems, established for intensive rotational grazing of *Urochloa brizantha* cv. BRS Piatã; (b) an integrated crop–livestock–forestry (ICLF) system—a shaded pasture system with an area of 12 ha, with 4 subsystems, established for intensive rotational grazing of *Urochloa brizantha* cv. BRS Piatã with eucalyptus trees (*Eucalyptus urograndis*, GG100 clone). The trees were arranged in single rows, in an east–west direction, with a spacing of 30 m between rows and 4 m between plants (83 trees/ha). The trees were, on average, 34.9 m tall and 38.1 cm in diameter at 1.30 m from the ground, resulting in an average reduction in photosynthetically active radiation (PAR) of 51%. PAR was recorded continuously at a height of 70 cm above ground level using SQ-301 linear quantum sensors (Apogee, Logan, UT, USA) in the FS and ICLF systems, and the reduction in radiation was obtained from the ratio between PAR ICLF/PAR FS [33].

### 2.5. Experimental Animals and Management

Forty young purebred bulls were used, 20 of which were Nelore (*Bos indicus*) (342.5 ± 36.6 kg BW; 16.9 ± 1.8 months old) and 20 Canchim (5/8 *Bos taurus* × 3/8 *Bos indicus*) (338.4 ± 39.8 kg BW; 19.1 ± 1.9 months old), with a minimum body condition score of 6.0, on a scale from 1 to 9 [34]. The animals had a known pedigree, health, and zootechnical history. The full-sun (FS) system consisted of 10 Nelore and 10 Canchim animals, which were allocated to non-shaded pastures. The integrated crop–livestock–forestry (ICLF) system had 10 Nelore and 10 Canchim animals, allocated to pasture areas with the availability of shade. The animals from both breeds were randomly distributed in the production systems.

The grazing cycles adopted in both production systems were 36 days long, with the animals rotating through the paddocks every 6 days and with a rest period of 30 days. Stocking rate adjustments were made using the “put and take” technique, in order to provide the animals with similar forage availability, regardless of the production system [35]. In both production systems, the animals had ad libitum access to water in automatic troughs and mineral supplementation in covered troughs located in the management center of the systems. The animals received the same nutritional and health management procedures.

### 2.6. Sensor-Based Behavioral Assessment

At the beginning of the experimental period, each animal received a collar with an electronic device, with an acoustic sensor and a triaxial accelerometer on board (C-Tech HealthyCow, CowMed Ltd., Rio Grande do Sul, Brazil). The details of the mesh network used to provide connectivity and cover all pasture areas have been previously described [27,36]. Electronic monitoring of individual behavior was carried out continuously throughout the experimental period, with activity patterns recorded every minute and with daily data outputs. The information was directly transferred wirelessly to data storage centers and from there to a single processing center [36]. The data were processed in a proprietary system (C-Manager, CowMed Ltd., Brazil), based on algorithms specifically designed to determine the activity of each animal. The recorded behaviors were categorized into movement, rumination, and resting. For each hour of evaluation, the duration of each behavior was calculated (minutes/hour) and transformed into a percentage of the time dedicated to the respective activity [37]. The results were presented for each hour, with the following intervals didactically considered as shifts: dawn (00:00 to 6:00 h), morning (6:00 to 12:00 h), afternoon (13:00 to 18:00 h), and night (18:00 to 00:00 h).

### 2.7. Direct Behavioral Assessment

Visual observations of the animals’ behavior were made monthly in the field, over two consecutive days, using a predefined ethogram. The assessments were carried out individually, with the animals identified numerically by non-toxic paint on their flanks, so as to allow observations at a greater distance than their flight zone, and to avoid interference from observers on the herd. Recordings were made every five minutes by a previously trained fixed team, using the instantaneous scan sampling technique [38] with 5 min intervals for recording behavioral parameters. Visual observations took place continuously from 8:00 to 16:00 h. For each hour, the duration of each behavior was calculated (minutes/hour) and transformed into a percentage of the time dedicated to the respective activity [37]. The results were presented for each hour and the visual observation period was didactically divided into morning (8:00 to 12:00 h) and afternoon (12:00 to 16:00 h) shifts, as adopted by [12].

The animals’ behavior was categorized by the posture and attitudes observed, namely: resting while lying, resting while standing, grazing, ruminating while lying, ruminating while standing. For the ICLF system, where shading was highly available, the time each animal spent in the sun or shade was also recorded. The location of the animal in the shade was considered when it had 50% or more of its body in the shaded area at the time of observation, as recommended [12,38]. The ethogram with the descriptors of the activities recorded and the positioning of the animals is presented in Supplementary Table S1. Additionally, in both systems, the frequency of water and mineral mixture intake was assessed, given by the number of times each animal went to the water trough or mineral mixture trough, respectively. The results for the frequency of mineral mixture intake and water intake were expressed as the average number of events per animal per shift.

### 2.8. Cortisol Dosage

Blood samples were taken once a month, on a day that did not coincide with the visual behavioral assessments, always in the morning (9:00 to 11:00 h). The animals were led at a walking pace using rational handling techniques to the corral adjacent to the grazing area. The samples were taken through venipuncture from the animals restrained in a chute, in 10 mL vacuum tubes, without anticoagulant. The samples were centrifuged at 4000 RPM for 15 min to separate the serum, fractionated into aliquots, and stored in polypropylene microtubes at −20 °C. Subsequently, serum cortisol concentrations were determined by radioimmunoassay using the Cortisol Immuchem Coated Tube kit (MP Biomedicals Diagnostics Division, Solon, OH, USA) [19]. The intra-assay coefficient was 8% and the inter-assay coefficient was 6%.

### 2.9. Statistical Analysis

A general linear model was used to evaluate the climatic variables and those related to the thermal environment of the production systems, including the fixed variables of Production System (FS or ICLF), Time (0, 1, 2, …23 h), a double interaction System–Time, as well as the random effects of days within the month of evaluation and residue. The variables related to behavior assessed by electronic sensors (rumination, movement, and resting) and by ethogram (resting while standing, resting while lying, grazing, ruminating while standing, ruminating while lying; in the sun or in the shade), expressed in minutes, were transformed into relative frequencies by dividing the time spent in each activity by 60 min (one hour). In this way, the relative frequencies were evaluated using a generalized linear mixed model with a logistic link function in order to relate the dependent variable to each behavior in the statistical model. The generalized linear mixed model included the fixed variables of Production System (FS or ICLF), Breed (Nelore or Canchim), Time (0, 1, 2, …23 h), double interactions System–Breed, System–Time, and Breed–Time, and a triple interaction System–Breed–Time, as well as the random effects of animal within batch and residue. For the variables of frequency of water intake and frequency of mineral mixture intake, a generalized linear mixed model with a logarithmic link function was adopted in order to relate the dependent variable of each behavior to the statistical model. In this case, records with zero values for a given behavior were excluded from the analysis and the model included the same fixed and random effects as mentioned above.

For the analysis of cortisol concentrations, a general linear mixed model was used which included the fixed effects of Production System (FS or ICLF), Month, Breed (Nelore or Canchim), double interactions System–Breed, System–Month, and Breed–Month, and a triple interaction System–Breed–Month, as well as the random effects of animal within batch and residue. The assumptions of the analysis of variance models (normality and homogeneity of residuals) were carried out simultaneously by means of Studentized conditional residual analyses. In the event of significant results for the fixed effects of Production System, Breed, Time, the double interactions, and the triple interaction, the LSD test was used, as appropriate, as a procedure for comparing the means, in order to maintain the set confidence level. All the analyses were carried out using the PROC MIXED or GLIMMIX procedures in the Statistical Analysis System, version 9.4 [39]. The significance level adopted for all analyses was 5%.

## 3. Results

### 3.1. Microclimate of Production Systems

**Figure 2:**
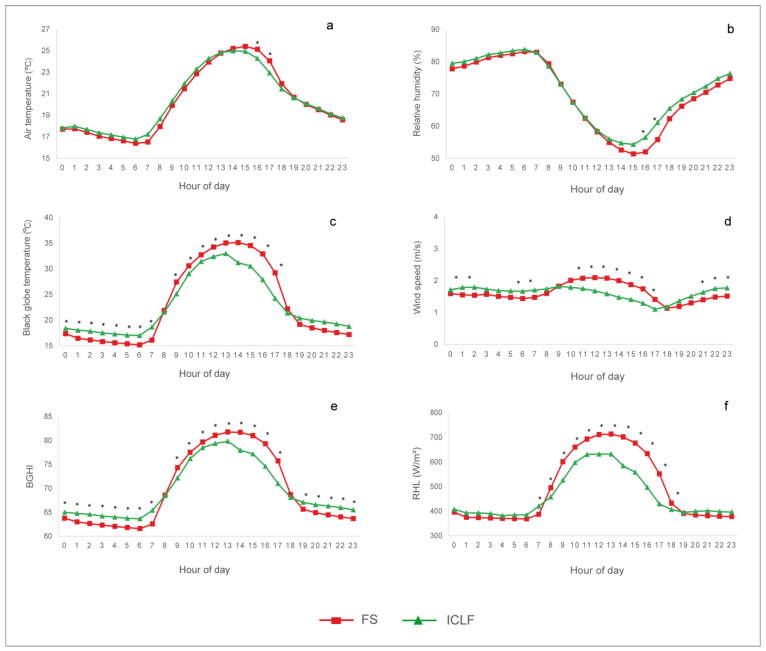
Hourly averages of (**a**) air temperature, (**b**) relative humidity, (**c**) black globe temperature, (**d**) wind speed, (**e**) black globe temperature and humidity index (BGHI), and (**f**) radiant heat load index (RHL) throughout the experimental period, in full-sun (FS) and integrated crop–livestock–forestry (ICLF) pastures. Asterisks indicate a significant difference between the production systems (*p* < 0.05).

The microclimatic characterization of the production systems during the experimental period is shown in Figure 2. The air temperature reached its highest values between 14:00 and 16:00 h, with a significant difference between the two production systems (*p* < 0.05). The average air temperature gradually decreased at night, from 18:00 h onwards, reaching values <21.0 °C in both systems. Relative humidity showed a significant difference in the afternoon, being lower in the FS system at 16:00 h (FS = 51.98 ± 1.02% vs. ICLF = 56.52 ± 1.02%; *p* < 0.05) and at 17:00 h (FS = 55.79 ± 1.02% vs. ICLF = 61.10 ± 1.02%; *p* < 0.05). The highest black globe temperature and BGHI values were recorded from 12:00 to 16:00 h, with a significant difference between the systems (*p* < 0.05). The FS system had a higher average BGHI than the ICLF system at 12:00 h (81.06 ± 0.36 vs. 79.38 ± 0.37), at 13:00 h (81.73 ± 0.36 vs. 79.86 ± 0.37), at 14:00 h (81.66 ± 0.36 vs. 77.96 ± 0.37), at 15:00 h (80.99 ± 0.36 vs. 77.21 ± 0.37), and at 16:00 h (79.32 ± 0.36 vs. 74.59 ± 0.37). RHL also showed a significant difference throughout the day, being higher in the FS system. The highest RHL value occurred at 13:00 h, both in the FS (712.60 ± 5.91 W/m²) and in the ICLF (632.20 ± 6.09 W/m^2^) systems.

### 3.2. Sensor-Based Behavioral Assessment

**Figure 3:**
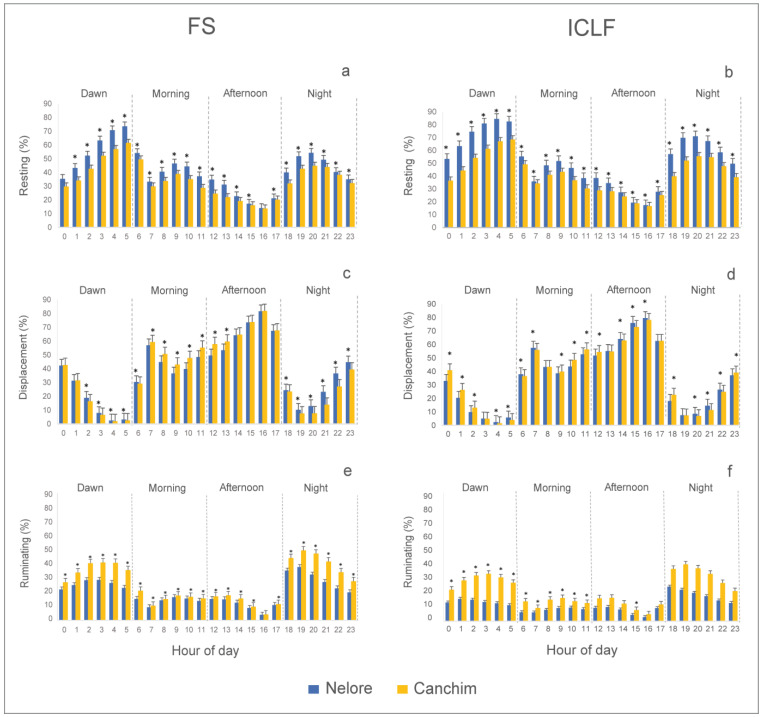
Behavior of young Nelore and Canchim bulls (*n* = 40) kept in full-sun pasture (FS; graphs **a**,**c**,**e**) or integrated crop–livestock–forestry (ICLF; graphs **b**,**d**,**f**) production systems, assessed by electronic monitoring and expressed as a percentage of time spent per hour (mean ± standard error). Asterisks indicate a significant difference between breeds within the hour (*p* < 0.05).

The attitudes recorded by electronic monitoring were presented as a percentage of time for each hour of recording (Figure 3) and were breed-dependent. There was a significant difference in resting time in all shifts. The Nelore bulls remained resting for longer than the Canchim, regardless of the production system (*p* < 0.001) at practically all the times analyzed. In turn, displacement was more pronounced in the morning and afternoon shifts, with more relevant and increasing wandering from 06:00 h onwards, peaking at 16:00 h, regardless of breed and production system. From 17:00 h onwards, there was a reduction in movement in both systems. There was a significant effect of breed on the displacement pattern (*p* = 0.01). Canchim bulls spent more time moving than Nelore (*p* < 0.001) in the morning and afternoon shifts, while Nelore spent more time moving than Canchim at night and in the early morning, especially in the FS system (*p* = 0.030). There was a significant effect of breed on the time devoted to rumination (*p* < 0.01), at all times of the day. The Canchim animals had longer rumination times than the Nelore, both in the FS and ICLF systems. The preferred times for rumination in both systems were concentrated in the evening and early morning shifts.

### 3.3. Direct Behavioral Assessment

The Nelore and Canchim bulls showed a significant difference in resting while lying, both in the morning and in the afternoon. In general, in both the FS and ICLF systems, the Nelore animals spent more time lying down than the Canchim (Table 1). However, in the ICLF system, from 8:00 to 11:00 h, the Canchim indicated a preference for lying down in the sun. In the system where there was no option to be in the shade, both the Nelore and Canchim animals spent a significant amount of time lying down from 10:00 to 13:00 h.

Most of the hours of resting while standing took place at the beginning of the day, from 8:00 to 10:00 h, for both breeds and systems. This feature was influenced by the breed (Table 2), with Nelore bulls spending more time standing than Canchim. In the FS system, Nelore bulls spent more time standing than Canchim at virtually any time of day. In the ICLF system, the differences were not significant between 11:00 and 13:00 h.

Grazing was the most notable activity during visual observations, with a significant difference between breeds at all assessed times (Table 3). Grazing time showed an increasing trend throughout the day (Supplementary Figure S1). Regardless of breed, grazing in the afternoons took up between 60 and 80% of the time for animals in the FS system, and between 45 and 60% of the time for animals in the ICLF system. The time spent grazing was significantly longer for Canchim bulls (*p* < 0.05), regardless of the production system. During grazing in the ICLF system, when there was a choice of remaining in the sun or in a shaded area, the Nelore bulls showed a preference for remaining in shaded areas, while the opposite was observed in the Canchim bulls. The preference of the Nelore and Canchim animals for carrying out different activities in the sun or shade, in the spaces made available within the integrated crop–livestock–forestry system, are shown in Supplementary Figure S2.

Animals of the Nelore and Canchim breeds differed significantly (*p* < 0.05) in the amount of time spent lying down to ruminate, especially in the ICLF system (Table 4). No behavioral pattern was detected that indicates a breed’s preference for lying down to ruminate in the sun or shade.

The activity of standing rumination was preferentially carried out in the morning. For this attribute, a distinctive pattern was noted between breeds (*p* < 0.05) at practically all times of the day, with the Nelore bulls spending more time ruminating standing up than Canchim bulls, regardless of the production system (Table 5). In general, the Nelore animals spent more time ruminating standing up in the sun than Canchim animals.

**Figure 4:**
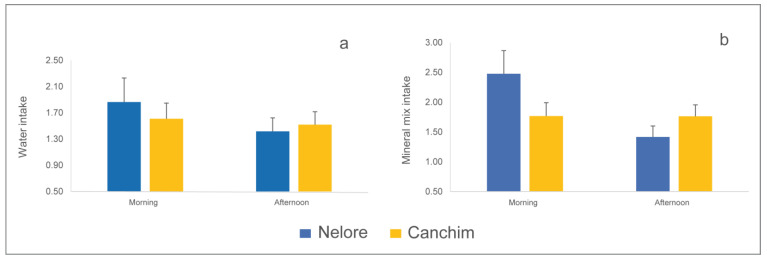
Number of visits (mean ± standard error) (**a**) to the water trough and (**b**) to the mineral mixture trough made by young Nelore and Canchim bulls (*n* = 40) kept in pasture production systems during the morning and afternoon shifts.

There was no significant difference between breeds in the frequency of water intake and the frequency of mineral mixture intake, either in the morning or in the afternoon (Figure 4). Although the Nelore bulls showed a certain preference for drinking water and ingesting mineral mixture in the morning, and the Canchim bulls did so more frequently in the afternoon, the two genotypes showed behavior with no significant difference that points to a distinction in this behavioral pattern between breeds during the shifts observed.

### 3.4. Cortisol Concentrations

**Figure 5:**
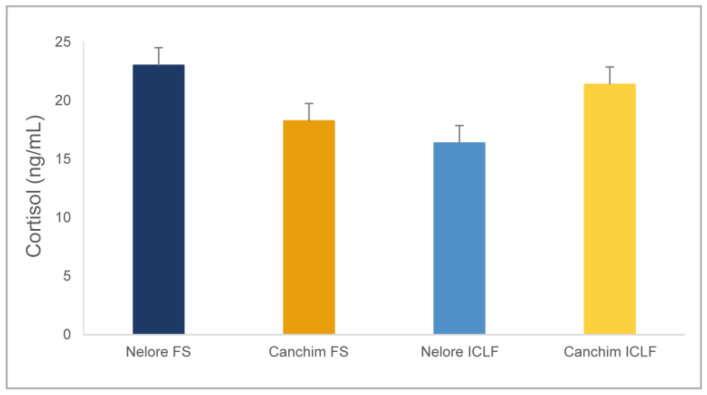
Mean (± standard error) serum cortisol concentrations of young Nelore and Canchim bulls (*n* = 40) kept in full-sun (FS) or integrated crop–livestock–forest (ICLF) pasture production systems during the experimental period.

Numerically, the dosage results were higher in the Nelore bulls from the FS and the Canchim from the ICLF system (Figure 5). However, there was no significant interaction between breeds and production systems (*p* = 0.082) that could confirm a significant effect of breed on serum cortisol concentrations, as a function of the different production systems studied.

## 4. Discussion

### 4.1. Microclimate of Production Systems

Beef production today faces a number of environmental challenges, most of which occur at the livestock level. It is therefore of particular interest to continuously develop strategies to make production systems more sustainable and animal-friendly [9]. In this context, the use of planted forests in pasture areas has been pointed out as a nature-based solution capable of increasing the diversity of agricultural systems [40], providing environmental services and offering greater thermal comfort to animals raised in tropical regions [41,42]. These positive effects were effectively demonstrated in this study, as the results showed an improvement in environmental indicators in the forested system (Figure 2). In both systems, the highest BGHI values were recorded at 13:00 h (FS = 81.73 ± 0.37 vs. ICLF = 79.86 ± 0.37; *p* < 0.05). The BGHI values observed in the FS system during the zenith characterized a condition of great thermal challenge, especially for taurine bulls raised on pasture, as previously described [43]. However, with the spatial configuration and tree density adopted, natural shading significantly reduced the BGHI between 2.3 and 5.9% in the afternoon shift.

In turn, radiant heat load is an indicator used to assess animal thermal comfort, as it incorporates the total radiation received by the black globe from all of the surrounding space [44] and characterizes the total radiation received by the animals [32]. In this study, the RHL was also lower in the ICLF system, as the presence of trees in the pastures reduced the radiant load by 11.2% at the time of the most intense radiation. As a result, the thermal condition of the afforested system was mitigated, with an unequivocal reduction in air temperature, which was about 2.0 °C lower at the hottest times compared to the FS system, although the trees kept relative humidity higher. This analysis provides evidence to support the positive effects of the integrated system on thermal comfort, an important element in promoting a better environment for the animals [45] and favoring the expression of their genetic potential [46].

### 4.2. Behavioral Assessment

One way to overcome the physical and time constraints associated with continuous visual observation of animals on pasture is to use electronic monitoring devices. Some sensors are capable of monitoring several attitudes simultaneously, are effective for presenting individual animal behavior continuously, and can be useful for monitoring health and welfare indicators [47]. In this study, the use of electronic sensors enabled uninterrupted assessment of the bulls throughout the day, during the entire experimental period. In an unprecedented way, we sought to study whether the genotype, which so largely determines anatomical and biochemical features, can also interact with the environment of production systems and influence the behavior of young bulls.

The time of day was a determining factor in the expression of daily activities. At first analysis, the distribution of the time spent by the experimental animals on movement, resting, and rumination, recorded uninterruptedly by electronic sensors, shows unmistakable characteristics of the behavior of a species with diurnal habits. According to [48], cattle raised on pasture, where feeding occurs spontaneously and is not induced by fixed feeding times, have feeding habits and time correlated to the times when light is most prevalent.

The animals spent more time resting and ruminating during the night and early morning, while locomotion occurred predominantly in the morning and afternoon in both systems (Figure 3). Similarly, ref. [27] recorded this behavioral trend in electronically monitored animals, but in adult bulls. The more significant displacement recorded in the morning and afternoon also corroborates studies in which cattle move for up to 12 h during the daytime hours, albeit not continuously, mainly to graze [49,50]. In turn, rumination time reached its lowest point in the late afternoon in both systems. This was possibly in response to the thermal challenge posed by the heat, represented by the gradual rise in BGHI and RHL from the morning until the afternoon, a phenomenon inherent to tropical climates. To avoid excessive heat accumulation under a heat challenge, cattle regulate their rumination time in an attempt to reduce the ruminal passage rate [26] and reduce the generation of endogenous heat resulting from rumen fermentation.

Regarding the genotypes, the breed component caused differences in behavior in both production systems. It was possible to observe that the Nelore bulls remained resting for longer than the Canchim in all shifts of the day and regardless of the production system. The fact that the Nelore animals spent more time at rest is highlighted by the direct visual observation records, which showed that the Zebu animals spent more time at rest than the composite animals. In the morning and afternoon shifts in both systems, the Canchim animals spent more time moving around than the Nelore animals. This finding, in conjunction with the results of visual observation, suggests that the more constant movement of Canchim animals may be related to greater exploration of the area and more time spent searching and reaching for feed. Therefore, there is evidence that the greater locomotion time of the Canchim animals during the day has an adaptive significance, as a response to the greater need to ingest forage. This assumption is reinforced by the fact that the Canchim animals had consistently longer rumination times when compared to the Nelore animals in both systems, at all times. Rumination time can be influenced by stress, both in a physiological condition [51], as well as when the animals are under environmental stress [52] or induced stress [53]. However, in this study, the difference in rumination time was not associated with cortisol as a stress biomarker, as there was no significant difference between breeds, regardless of the production system evaluated (Figure 5).

Direct visual observations were made to add information to that captured by the sensors, in order to provide a more detailed understanding of the animals’ behavior. The use of the ethogram by visual observation is more flexible in terms of the characteristics of the records and provides specific elements about some distinct attitudes and the spatial positioning of the animals, which cannot yet be parameterized by electronic sensor algorithms. Thus, the two monitoring techniques used are complementary and guarantee more reliable records of animal behavior, as they are based on records of different events.

Considering the ethogram adopted, grazing was the first and most frequent activity carried out during the day, regardless of breed (Table 3); a result in line with that postulated by [54]. According to [55], between 65 and 100% of grazing activity takes place between 6:00 h and 19:00 h. In a more recent study, ref. [56] reported that the longest grazing time for cattle is from 7:00 to 14:00 h. These definitions are similar to the results obtained, which were even more specific and indicated a concentration of grazing from 12:00 to 16:00 h. In this study, grazing took between 35.4 and 79.7% of the animals’ time, regardless of breed, in the FS system. In the ICLF system, the animals spent between 33.7 and 47.3% of their time grazing in the sun and between 10.0 and 14.3% grazing in the shade. This can be explained by the heterogeneity of forage availability within the ICLF system, with a greater supply of forage in the non-shaded fractions [57], since photosynthetically active radiation is reduced under the tree canopies.

The Canchim animals spent more time grazing in the full-sun system, at all times of the day, when compared to the Nelore animals. The greater nutritional demand leads to greater intensity in the search for feed [58], which increases the animals’ displacement in the pastures. In the ICLF system, the Canchim animals spent more time grazing than the Nelore in the morning, but only when occupying the shaded areas of the pasture. From 11:00 h onwards, the Canchim animals spent more time grazing than the Nelore, but in the non-shaded areas of the pasture, while, on the other hand, the Nelore animals spent more time grazing in the shade. However, the fact that the Canchim bulls spent more time in the unshaded areas during the hottest times did not lead to heat stress or even greater stress, compared to the Nelore bulls kept in the same production system.

This finding is supported by the results observed for serum cortisol concentration (Figure 5), whose normal range in cattle varies between 5.0 and 12.4 ng/mL [59,60]. Although not necessarily painful, certain handling practices, such as restraint in a chute, might result in elevated cortisol secretion [61]. However, blood cortisol levels are not affected if the sample is collected within a few minutes after the animal is restrained [62]. Hormonal patterns in cattle vary cyclically approximately every 24 h, a process referred to as diurnal rhythm or circadian cycle. For this reason, the glucocorticoid response to stress is immediate, with cortisol concentrations rising rapidly, reaching values many times higher than normal within minutes [63]. Despite being a composite breed and, in theory, more subject to heat stress than zebu, the adaptive phenotypic characteristics of the Canchim animals, such as dense coat made up of short, light hair [17] may have favored their thermoregulation. Such coat attributes favor partial reflection of solar radiation and minimize heat absorption, benefiting thermal exchange with the external environment and homeothermy [64]. Another possible explanation for the longer occupation time of the non-shaded areas by the Canchim animals could be associated with the social hierarchy within the experimental groups. It is known that in silvopastoral systems, dominant cattle prefer to graze in the shade at the hottest times, pushing subordinate animals back to less favorable microclimate locations [65]. However, as agonistic interactions between individuals were not assessed in this study, this connection with the hierarchy could not be determined.

Some studies show that the lying-down behavior adopted by cattle is common in situations of lower thermal comfort, especially during the hottest periods. This is a behavioral strategy induced by the environment, in which animals aim to voluntarily slow down their movements and reduce the generation of endogenous heat, which has direct benefits for homeothermy. For this reason, the animals of both genotypes had the interval from 10:00 to 13:00 h as their preferred time for lying down in the FS system, in an obvious behavioral response to the stimulus generated by the higher temperature and radiation (Table 1), as indicated by the BGHI and RHL. This behavioral response was also reported by [52], indicating that cattle tend to reduce rumination time and increase resting at higher temperatures in an attempt to maintain or re-establish their thermal balance. When the animals had the option of being in the sun or shade, a situation only possible in the system with the presence of the trees, the Nelore spent more time resting while lying in the shade, from 8:00 to 14:00 h, while the Canchim spent more time resting while lying in the sun, in the morning shift.

In turn, resting while in the standing position reduces the generation of endogenous heat, allows thermal energy to be dissipated by convective route [66] and facilitates thermoregulation. This may explain the increase in time resting while standing in Nelore animals (Table 2), as a sign of a behavior acquired through conditioning and related to the preservation of homeothermy. The time spent ruminating while lying down showed an erratic pattern, with no breed preponderance (Table 4). However, the Nelore bulls spent more time ruminating standing up than the Canchim (Table 5), a result which may be due to the greater time spent moving around at night and in the early hours of the morning for the Nelore animals, times when they should be resting or ruminating, based on the circadian rhythm of bovine behavior.

Water is the most important natural resource for production and survival. It plays an important role in physiological processes and is co-responsible for maintaining the internal body temperature of animals [67]. In regions with hot climates, it is necessary to highlight the importance of climatic factors in drinking, as the greater consumption of water is due to the need to cool the body and maintain electrolyte balance [68]. Although there was no significant difference between shifts, the animals of both breeds had a numerically higher frequency of visits to the water trough and mineral mixture trough in the morning (Figure 4). These events followed the upward trend in air temperature and solar radiation recorded during the day. Since the experimental animals of both genotypes had a similar average age and the same nutritional management, the findings suggest that the frequency of drinking and searching for mineral mixture is associated with maintaining electrolyte balance, which is essential for normal thermolysis [27]. Although they were not evaluated in this study, the two main responses of cattle to heat are sweating and increased respiratory rate, which have the side effect of reducing body reserves of water and electrolytes, with the need for rapid replacement [69]. The frequency of water and mineral mixture intake was similar for both breeds and was not affected by the production systems. These results partially agree with those observed for beef suckling cows, which did not have their frequency of seeking mineral mix affected, but showed a 23% reduction in visits to the water trough when raised in shaded pasture systems [12].

## 5. Conclusions

The presence of the tree component in an integrated crop–livestock–forestry system in a tropical environment improved the microclimatic conditions of the pastures, increased thermal comfort, and did not cause any negative distortions in the pattern of daytime habits of the cattle. Notably, the availability of natural shading had an impact on the distribution of time spent grazing, resting, and ruminating. Genetic differences between zebu and composite animals contributed to the expression of behavior, which was dependent on the production system adopted. The Canchim bulls were more active when moving and grazing, while the Nelore bulls spent more time resting; shown to be an adaptive strategy derived from environmental stimuli. The frequency of water and mineral mixture intake did not differ between genotypes, regardless of the production system.

Thus, the results of this study can help farmers make more assertive decisions when planning the implementation of pasture-based production systems, where the incorporation of trees through forest planting is highly recommended. This recommendation is based on the better thermal comfort indicators observed on afforested pastures. In addition, a comprehensive knowledge of the natural behavior of cattle kept on pasture can help in the daily management of the animals. This allows for better planning in determining the number of animals and breed factors for forming lots and in setting schedules for management activities. In this way, harmful interactions between animals and between animals and the environment are avoided; the risk of accidents, wasted inputs and economic losses is reduced; animal welfare is improved; and more sustainable and efficient livestock management practices are promoted.

## Figures and Tables

**Table 1:** Time spent resting while lying by young bulls (*n* = 40) kept in full-sun (FS) or integrated crop–livestock–forestry (ICLF) pasture systems. Results expressed as a percentage per hour (mean ± standard error).

Time	Positioning	FS				ICLF			
		Nelore	Canchim	RMSE	*p*-Value	Nelore	Canchim	RMSE	*p*-Value
8:00–9:00 h	In the sun	2.85	2.61	0.252	0.94	2.24 ^b^	5.80 ^a^	0.252	0.01
	In the shade	-	-			2.87 ^b^	5.05 ^a^	0.658	0.01
9:00–10:00 h	In the sun	13.33	12.95	0.252	0.94	7.98 ^b^	10.47 ^a^	0.252	0.01
	In the shade	-	-			7.87 ^a^	3.83 ^b^	0.658	0.01
10:00–11:00 h	In the sun	26.57	26.11	0.252	0.94	4.83 ^b^	6.42 ^a^	0.252	0.01
	In the shade	-	-			5.67 ^a^	3.92 ^b^	0.658	0.01
11:00–12:00 h	In the sun	26.26 ^a^	21.29 ^b^	0.252	0.04	6.86	7.44	0.252	0.99
	In the shade	-	-			7.24 ^a^	4.57 ^b^	0.658	0.01
12:00–13:00 h	In the sun	15.95	16.85	0.252	0.94	10.28 ^a^	7.38 ^b^	0.252	0.01
	In the shade	-	-			7.30 ^a^	5.31 ^b^	0.658	0.01
13:00–14:00 h	In the sun	15.66 ^a^	10.58 ^b^	0.252	0.04	8.10 ^a^	6.28 ^b^	0.252	0.01
	In the shade	-	-			6.28 ^a^	4.57 ^b^	0.658	0.01
14:00–15:00 h	In the sun	9.99 ^a^	5.82 ^b^	0.252	0.04	2.54	2.31	0.252	0.99
	In the shade	-	-			4.03	4.98	0.658	0.99
15:00–16:00 h	In the sun	4.37 ^b^	5.62 ^a^	0.252	0.04	1.37 ^b^	3.30 ^a^	0.252	0.01
	In the shade	-	-			0.65 ^b^	2.58 ^a^	0.658	0.01

(-) Not applicable. ^(a,b)^ Different letters on the line within the same production system indicate significant differences between breeds at the same time (*p* < 0.05).

**Table 2:** Time spent resting while standing by young bulls (*n* = 40) kept in full-sun (FS) or integrated crop–livestock–forestry (ICLF) pasture systems. Results expressed as a percentage per hour (mean ± standard error).

Time	Positioning	FS				ICLF			
		Nelore	Canchim	RMSE	*p*-Value	Nelore	Canchim	RMSE	*p*-Value
8:00–9:00 h	In the sun	35.49 ^a^	24.44 ^b^	0.235	0.01	17.82 ^a^	12.63 ^b^	0.235	0.01
	In the shade	-	-			14.22 ^a^	9.37 ^b^	0.690	0.01
9:00–10:00 h	In the sun	33.55 ^a^	28.88 ^b^	0.235	0.01	8.60	8.42	0.235	0.98
	In the shade	-	-			7.03	7.84	0.690	0.98
10:00–11:00 h	In the sun	16.64 ^a^	11.72 ^b^	0.235	0.01	9.63 ^a^	7.03 ^b^	0.235	0.01
	In the shade	-	-			5.56	5.70	0.690	0.98
11:00–12:00 h	In the sun	14.43	15.46	0.235	0.68	8.03	8.48	0.235	0.98
	In the shade	-	-			3.09	3.76	0.690	0.98
12:00–13:00 h	In the sun	12.13 ^a^	7.37 ^b^	0.235	0.01	4.51	4.90	0.235	0.98
	In the shade	-	-			3.57	2.96	0.690	0.98
13:00–14:00 h	In the sun	14.17 ^a^	8.97 ^b^	0.235	0.01	3.90	3.84	0.235	0.98
	In the shade	-	-			5.50 ^a^	3.60 ^b^	0.690	0.01
14:00–15:00 h	In the sun	14.23	13.33	0.235	0.68	4.08 ^b^	6.77 ^a^	0.235	0.01
	In the shade	-	-			7.48 ^a^	6.37 ^b^	0.690	0.01
15:00–16:00 h	In the sun	9.82 ^a^	7.58 ^b^	0.235	0.01	1.61 ^b^	3.73 ^a^	0.235	0.01
	In the shade	-	-			4.85 ^a^	3.48 ^b^	0.690	0.01

(-) Not applicable. ^(a,b)^ Different letters on the line within the same production system indicate significant differences between breeds at the same time (*p* < 0.05).

**Table 3:** Time spent grazing by young bulls (*n* = 40) kept in full-sun (FS) or integrated crop–livestock–forest (ICLF) pasture systems. Results expressed as percentages per hour (mean ± standard error).

Time	Positioning	FS				ICLF			
		Nelore	Canchim	RMSE	*p*-Value	Nelore	Canchim	RMSE	*p*-Value
8:00–9:00 h	In the sun	53.26 ^b^	64.28 ^a^	0.323	0.01	14.42	14.42	0.323	0.99
	In the shade	-	-			14.80 ^b^	19.95 ^a^	1.227	0.01
9:00–10:00 h	In the sun	40.50 ^b^	44.27 ^a^	0.323	0.01	21.20 ^a^	18.36 ^b^	0.323	0.01
	In the shade	-	-			15.02 ^b^	17.20 ^a^	1.227	0.01
10:00–11:00 h	In the sun	35.45 ^b^	44.60 ^a^	0.323	0.01	33.78	36.23	0.323	0.99
	In the shade	-	-			12.12 ^b^	12.30 ^a^	1.227	0.01
11:00–12:00 h	In the sun	40.57 ^b^	49.80 ^a^	0.323	0.01	38.19 ^b^	42.69 ^a^	0.323	0.01
	In the shade	-	-			11.75 ^a^	11.27 ^b^	1.227	0.01
12:00–13:00 h	In the sun	56.42 ^b^	61.62 ^a^	0.323	0.01	33.56 ^b^	47.37 ^a^	0.323	0.01
	In the shade	-	-			13.70 ^a^	10.06 ^b^	1.227	0.01
13:00–14:00 h	In the sun	61.82 ^b^	72.89 ^a^	0.323	0.01	35.11 ^b^	40.99 ^a^	0.323	0.01
	In the shade	-	-			14.31 ^a^	12.94 ^b^	1.227	0.01
14:00–15:00 h	In the sun	63.78 ^b^	68.13 ^a^	0.323	0.01	27.46 ^b^	33.71 ^a^	0.323	0.01
	In the shade	-	-			22.87 ^a^	19.05 ^b^	1.227	0.01
15:00–16:00 h	In the sun	76.23 ^b^	79.76 ^a^	0.323	0.01	25.76	27.59	0.323	0.99
	In the shade	-	-			36.13 ^a^	31.09 ^b^	1.227	0.01

(-) Not applicable. ^(a,b)^ Different letters on the line within the same production system indicate significant differences between breeds at the same time (*p* < 0.05).

**Table 4:** Time spent ruminating while lying down by young bulls (*n* = 40) kept in full-sun (FS) or integrated crop–livestock–forestry (ICLF) pasture systems. Results expressed as a percentage per hour (mean ± standard error).

Time	Positioning	FS				ICLF			
		Nelore	Canchim	RMSE	*p*-Value	Nelore	Canchim	RMSE	*p*-Value
8:00–9:00 h	In the sun	1.07	1.20	0.808	0.45	0.67	1.05	0.808	0.18
	In the shade	-	-			0.00	2.92	1.317	0.18
9:00–10:00 h	In the sun	2.78	2.71	0.808	0.45	3.56	3.89	0.808	0.18
	In the shade	-	-			11.80 ^a^	8.48 ^b^	1.317	0.01
10:00–11:00 h	In the sun	6.57	5.71	0.808	0.45	4.02 ^b^	5.66 ^a^	0.808	0.01
	In the shade	-	-			5.36 ^b^	7.53 ^a^	1.317	0.01
11:00–12:00 h	In the sun	5.67 ^a^	3.79 ^b^	0.808	0.01	3.42 ^b^	7.70 ^a^	0.808	0.01
	In the shade	-	-			11.55 ^a^	5.90 ^b^	1.317	0.01
12:00–13:00 h	In the sun	4.75	4.52	0.808	0.45	12.49 ^a^	9.74 ^b^	0.808	0.01
	In the shade	-	-			5.62 ^b^	9.34 ^a^	1.317	0.01
13:00–14:00 h	In the sun	2.01	1.93	0.808	0.45	6.64 ^b^	12.13 ^a^	0.808	0.01
	In the shade	-	-			7.78	9.16	1.317	0.18
14:00–15:00 h	In the sun	3.47	3.60	0.808	0.45	3.62 ^a^	0.98 ^b^	0.808	0.01
	In the shade	-	-			10.11	10.03	1.317	0.18
15:00–16:00 h	In the sun	2.07	1.73	0.808	0.45	2.23	2.97	0.808	0.18
	In the shade	-	-			6.04	5.97	1.317	0.18

(-) Not applicable. ^(a,b)^ Different letters on the line within the same production system indicate significant differences between breeds at the same time (*p* < 0.05).

**Table 5:** Time spent ruminating while standing by young bulls (*n* = 40) kept in full-sun (FS) or integrated crop–livestock–forestry (ICLF) pasture systems. Results expressed as percentages per hour (mean ± standard error).

Time	Positioning	FS				ICLF			
		Nelore	Canchim	RMSE	*p*-Value	Nelore	Canchim	RMSE	*p*-Value
8:00–9:00 h	In the sun	3.17 ^a^	1.97 ^b^	0.307	0.01	1.43 ^a^	0.43 ^b^	0.307	0.01
	In the shade	-	-			4.06	2.92	0.562	0.99
9:00–10:00 h	In the sun	4.67	4.33	0.307	0.68	1.52	1.12	0.307	0.99
	In the shade	-	-			3.44	2.42	0.562	0.99
10:00–11:00 h	In the sun	5.78 ^a^	2.87 ^b^	0.307	0.01	3.75	3.20	0.307	0.99
	In the shade	-	-			2.65	1.64	0.562	0.99
11:00–12:00 h	In the sun	2.35	2.05	0.307	0.68	3.48 ^a^	0.56 ^b^	0.307	0.01
	In the shade	-	-			0.73	0.38	0.562	0.01
12:00–13:00 h	In the sun	1.56	1.48	0.307	0.68	1.09 ^a^	0.16 ^b^	0.307	0.01
	In the shade	-	-			0.14	0.51	0.562	0.99
13:00–14:00 h	In the sun	0.89 ^a^	0.47 ^b^	0.307	0.01	0.62	0.64	0.307	0.99
	In the shade	-	-			1.15	0.88	0.562	0.99
14:00–15:00 h	In the sun	1.68	1.86	0.307	0.68	0.35 ^b^	0.79 ^a^	0.307	0.01
	In the shade	-	-			0.67	0.69	0.562	0.99
15:00–16:00 h	In the sun	1.47 ^a^	0.84 ^b^	0.307	0.01	0.45	0.33	0.307	0.99
	In the shade	-	-			0.27	0.38	0.562	0.99

(-) Not applicable. ^(a,b)^ Different letters on the line within the same production system indicate significant differences between breeds at the same time (*p* < 0.05).

## Data Availability

The data are available upon request and institutional approval.

## Supplementary Materials

Supplementary material to this article can be found online at https://www.mdpi.com/article/10.3390/ani14060944/s1. Table S1. Ethogram with behavior and positioning descriptors used in direct observations to evaluate the behavior of cattle kept on pasture production systems. Figure S1. Grazing time spent by young Nelore and Canchim bulls (% of time, each hour) performing different activities in an integrated crop–livestock–forestry system, when spaces in the sun or shade were available in the pastures. Figure S2. Preference of young Nelore and Canchim bulls (% of time, each hour) for carrying out different activities in a crop–livestock–forest integration system, when spaces in the sun or shade were available in the pastures. (**a**): ruminating while standing (%). (**b**): ruminating while lying (%). (**c**): resting while standing (%). (**d**): resting while lying (%). (**e**): grazing (%). Refs. [70,71,72,73] are cited in Supplementary Materials.
