# Supplementary material for: Differences in the Behavioral Parameters of Young Zebu and Composite Bulls Kept on Non-Forested or in Integrated Crop–Livestock–Forestry Systems
Source: Animals (Basel). 2024 Mar 19;14(6):944. doi: 10.3390/ani14060944 (PMC10967639; doi:10.3390/ani14060944)
Supplement: Supplementary file 1 [file animals-14-00944-s001.zip › animals-2831162-supplementary.pdf]

Supplementary Material

Differences in the Behavioral Parameters of Young Zebu and Composite Bulls Kept on Non-Forested or in Integrated Crop–Livestock–Forestry Systems

Mariana Jucá Moraes [1], Erick Fonseca de Castilho [2], Júlio Cesar de Carvalho Balieiro [3], Alberto Carlos de Campos Bernardi [4], Andréa do Nascimento Barreto [1], Lívia Ferreira Pinho [1], Giovanna Galhardo Ramos [3], Gabriela Novais Azevedo [5], Letícia Krügner Zanetti [5] and Alexandre Rossetto Garcia [1,3,4,*]

[1] Institute of Veterinary Medicine, Federal University of Pará, Av. dos Universitários, s/n, Castanhal 68746-360, PA, Brazil; marianajmoraes15@gmail.com (M.J.M.); andreadnb91@gmail.com (A.d.N.B.); liviapinho30@gmail.com (L.F.P.)
[2] Institute of Animal Health and Production, Federal Rural University of the Amazon, Av. Perimetral, Belém 66077-830, PA, Brazil; erick.castilho@ufra.edu.br
[3] School of Veterinary Medicine and Animal Science, University of São Paulo, Av. Duque de Caxias Norte, Pirassununga 13630-520, SP, Brazil; balieiro@usp.br (J.C.d.C.B.); giiovannagalhardo@gmail.com (G.G.R.)
[4] Brazilian Agricultural Research Corporation, Embrapa Southeast Livestock, Rod. Washington Luiz, São Carlos 13560-970, SP, Brazil; alberto.bernardi@embrapa.br
[5] Central University Centre of São Paulo, R. Miguel Petroni, São Carlos 13563-470, SP, Brazil; gabi.nazevedo@hotmail.com (G.N.A.); letyzanettii@gmail.com (L.K.Z.)
* Correspondence: alexandre.garcia@embrapa.br

Journal: Animals - Special Issue "Beef Cattle Production and Management" (ISSN 2076-2615)

Supplementary Table S1. Ethogram with behavior and positioning descriptors used in direct observations to evaluate the behavior of cattle kept on pasture production systems.

Activity[£], Position[¥] and Positioning[€]	Description	Reference
Grazing[£]	The animal carries out the stages of selecting and grasping the forage, chewing and swallowing.	Pereira et al. (2005)
Rumination[£]	The animal performs jaw movements, chewing and swallowing a rumen bolus, without grazing.	Baggio et al. (2008); Poulopoulou et al. (2019)
Resting[£]	The animal does not move around in search of feed, nor does it graze or ruminate.	Phillips and Rind (2002)

Standing[¥]	The animal stands in a quadrupedal position, with its limbs extended and its abdomen completely off the ground.	Vizzotto et al. (2015)
Lying down[¥]	The animal lays in sternal decubitus, with its abdomen/thorax in contact with the ground.	Vizzotto et al. (2015)
In the sun[€]	Use of the sun is considered when the animal has 50% or more of its body in the area exposed to the sun.	Giro et al. (2019)
In the shade[€]	The use of shade is considered when the animal has 50% or more of its body in the shaded area.	Giro et al. (2019); Kendall et al. (2006)

Baggio, C., Carvalho, P.C.F., Silva, J.L.S., Rocha, L.M., Bremm, C., Santos, D.T., Monteiro, A.L.G., 2008. Padrões de uso do tempo por novilhos em pastagem consorciada de azevém anual e aveia preta. Revista Brasileira de Zootecnia 37, 1912-1918. https://doi.org/10.1590/S1516-35982008001100002.

Giro, A., Pezzopane, J.R.M., Junior, W.B., Pedroso, A.F., Lemes, A.P., Botta, D., Romanello, N., Barreto, A.N., Garcia, A.R., 2019. Behavior and body surface temperature of beef cattle in integrated crop-livestock systems with or without tree shading. Science of the Total Environment 684, 587-596. https://doi.org/10.1016/j.scitotenv.2019.05.377.

Kendall, P.E., Nielsen, P.P., Webster, J.R., Verkerk, G.A., Littlejohn, R.P., Matthews, L.R., 2006. The effects of providing shade to lactating dairy cows in a temperate climate. Livestock Science 103, 148-157. https://doi.org/10.1016/j.livsci.2006.02.004.

Pereira, L.M.R., Fischer, V., Moreno, C.B., Pardo, M.P., Gomes, J.F., Monks, P.L., 2005. Comportamento ingestivo diurno de novilhas Jersey em pastejo recebendo diferentes suplementos. Revista Brasileira Agrociência 11, 453-459. https://doi.org/10.18539/cast.v11i4.1285.

Phillips, C.J., Rind, M.I., 2002. The effects of social dominance on the production and behavior of grazing dairy cows offered forage supplements. Journal of Dairy Science 85, 51-59. https://doi.org/10.3168/jds.S0022-0302(02)74052-6.

Poulopoulou, I., Lambertz, C., Gauly, M., 2019. Are automated sensors a reliable tool to estimate behavioural activities in grazing beef cattle? Applied Animal Behaviour Science 216, 15. https://doi.org/10.1016/j.applanim.2019.04.009.

Vizzotto, E.F., Fischer, V., Thaler Neto, A., Abreu, A.S., Stumpf, M.T., Werncke, D., Schmidt, F.A., McManus, C.M., 2015. Access to shade changes behavioral and physiological attributes of dairy cows during the hot season in the subtropics. Animal 9, 1559-1566. https://doi.org/10.1017/S1751731115000877.

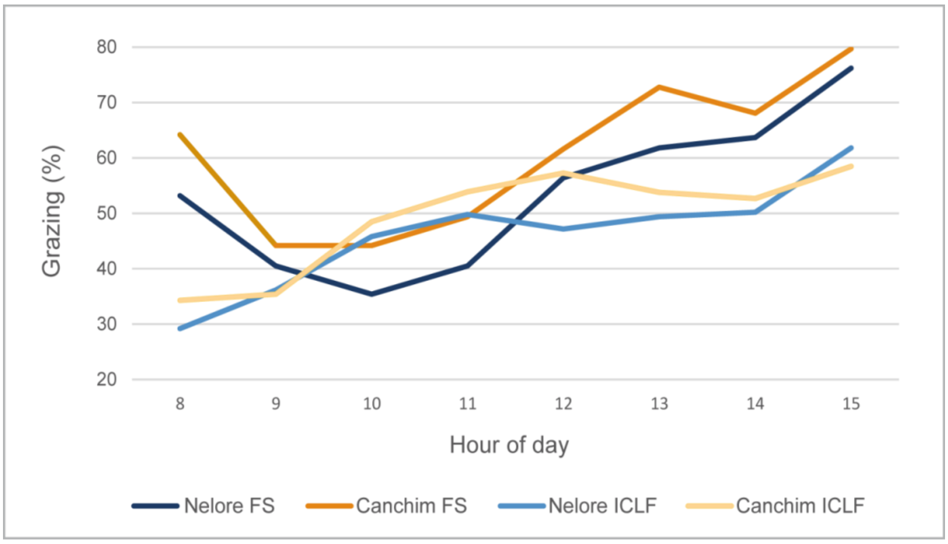

Supplementary Figure S1. Grazing time spent by young Nelore and Canchim bulls (% of time, each hour) performing different activities in an integrated crop-livestock-forestry system, when spaces in the sun or shade were available in the pastures.

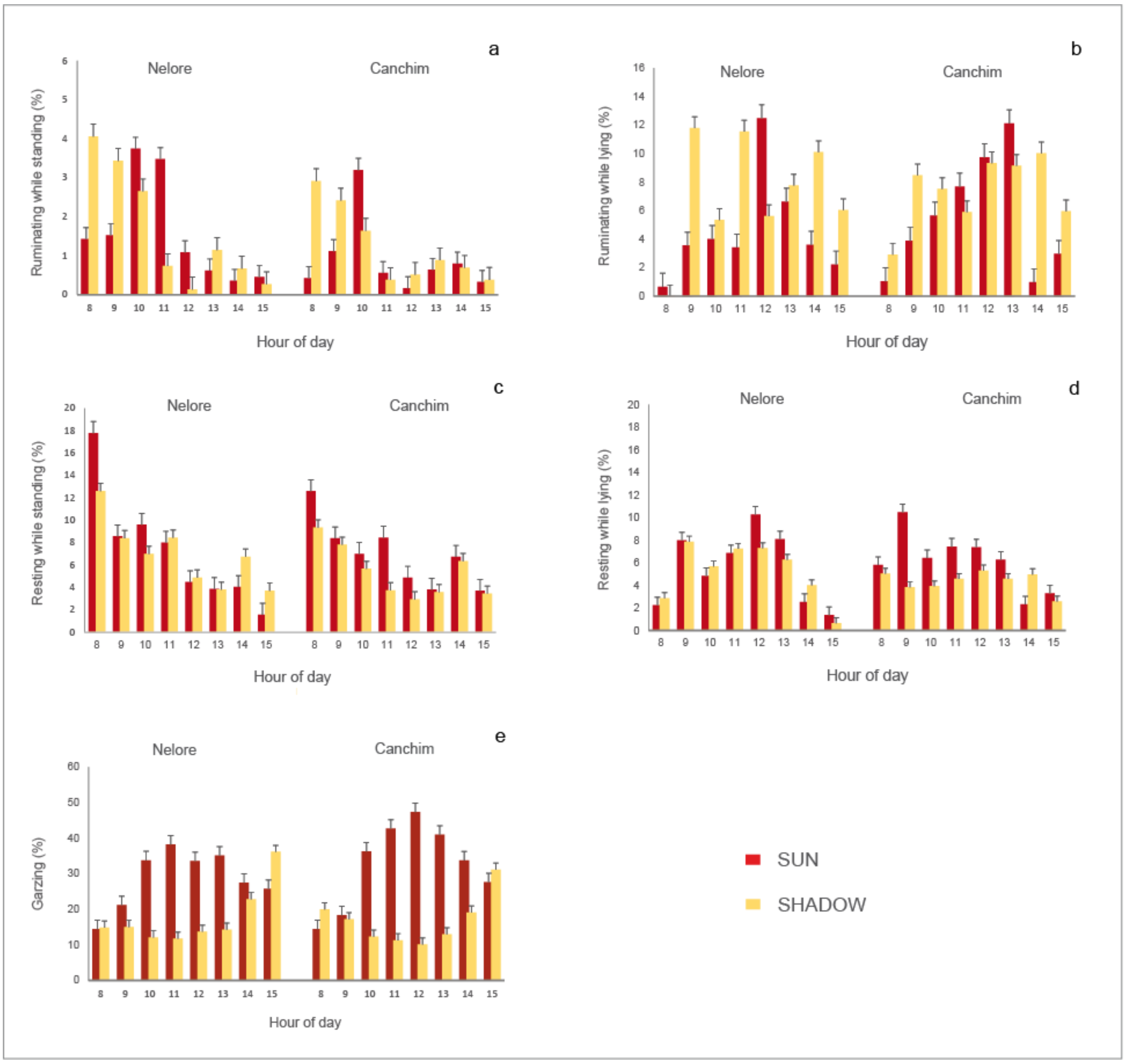

Supplementary Figure S2. Preference of young Nelore and Canchim bulls (% of time, each hour) for carrying out different activities in a crop-livestock-forest integration system, when spaces in the sun or shade were available in the pastures. (a): ruminating while standing (%). (b): ruminating while lying (%). (c): resting while standing (%). (d): resting while lying (%). (e): grazing (%).
